# Characterization and source apportionment of heavy metal contamination in agricultural soils in the complex genesis region of western Yunnan

**DOI:** 10.1038/s41598-025-16520-5

**Published:** 2025-09-01

**Authors:** Yingmei Li, Sheng Wang, Xiaoyan Shang, Hongyin Zhou, Jianyang He, Wen Chen, Lijuan Wang, Xiangmei Zhao, Li Bao, Naiming Zhang

**Affiliations:** 1 College of Resources and Environment, Yunnan Agricultural University, Kunming, 650201 China; 2 School of Chemistry and Geographical Sciences, Chuxiong Normal University, Chuxiong, 675000 China; 3 College of Biology and Chemistry, Pu’er University, Pu’er, 665000 China; 4 Yunnan Soil Fertility and Pollution Remediation Engineering Research Center, Kunming, 650201 China; 5 School of Agriculture and Life Sciences, Zhaotong University, Zhaotong, 657000 China

**Keywords:** High geological background, Heavy metal contamination, Cropland soil, Source analysis, Environmental sciences, Sustainability

## Abstract

The genesis of heavy metal contamination in arable soils is complex, and scientifically identifying risks and precisely analyzing contamination sources are essential for safely using contaminated arable land. In this study, we systematically evaluated the pollution characteristics of Cu, Zn, As, Hg, Cd, Pb, Ni, and Cr in soil, and then applied the APCS-MLR and PMF models to jointly analyze pollution sources and their contributions. The results showed that the concentrations of the eight heavy metals were significantly higher than the background values for soils in Yunnan Province, exhibiting clear spatial heterogeneity. The overall pollution level ranged from mild to severe, with Cd and Pb being the most critical contaminants. Four major pollution sources (industrial transportation, parent material, agriculture, and mining) were identified through the dual modeling approach. The results of both models corroborated each other, and the accuracy of the analysis was significantly improved compared to using a single method. This study not only provides a scientific basis for the safe utilization of contaminated arable land in western Yunnan, an area with a complex genesis of soil contamination, but also offers a generalized framework for source analysis in areas affected by geological-anthropogenic composite pollution.

## Introduction

Soil is an important resource for safeguarding human survival and development, and it is closely related to food security and ecological sustainability^1,2^. With the rapid development of urbanization and industrialization, soil heavy metal pollution has become a serious environmental problem^3,4^. In China, the limited per capita availability of arable land has led to the continued use of soils contaminated with heavy metals for agricultural production, in order to maintain food supply and meet land-use demands^5^. According to statistics, about 19.4% of China’s arable land has varying degrees of heavy metal contamination^6,7^. Because of its unique geologic background^8^ and the impact of human activities, the western Yunnan region has become a high-risk area for soil heavy metal pollution^9,10^. The complex causes of the soil heavy metal pollution in this region have made pollution tracing more challenging and pollution management more complex^11–13^. Compared with other high-background regions such as Greece and India, western Yunnan exhibits more intricate pollution characteristics due to its highly variable parent materials, intensive geological activity, and the overlapping impacts of mining, agriculture, and urbanization. However, current studies focusing on this region remain limited, leaving a gap in source identification research under such complex conditions.Therefore, it is crucial to investigate the risks and sources of heavy metal pollution in arable land in this region. Such research provides a scientific basis for effective source control and targeted pollution management.

Spatial heterogeneity is a key feature of soil heavy metal pollution. Numerous studies have demonstrated that spatial variation is influenced by both natural factors—such as geological background, topography, and landforms—and anthropogenic activities^14^. Chen et al.^15^ evaluated the spatial distribution characteristics and contamination levels of soil heavy metals in various administrative regions in China, and found that their concentrations in cultivated land in the south were significantly higher than those in the north. Hu et al.^16^ demonstrated that concentrations of multiple heavy metals, with the exception of Hg, surpassed local background values, indicating that agricultural activities played a predominant role in the enrichment of heavy metals in surface soils. A variety of technical tools and modeling analysis methods have been widely used in source analysis of soil heavy metal pollution^17–20^. Among them, the absolute principal component scores-multiple linear regression (APCS-MLR) model, which combines principal component analysis (PCA) and multiple linear regression (MLR), is able to quantify the contributions of different pollution sources under simple pollution scenarios^4^. The positive matrix factorization (PMF) (orthogonal minimum matrix factor) model has been widely used in soil heavy metal pollution research in recent years due to its advantage of evaluating the uncertainty of the source components^21^. For example, in studies focusing on coking industry regions, the PMF model effectively attributed the majority of soil heavy metal pollution to industrial emissions^22^. Lui et al.^23^ combined the results of the APCS-MLR and PMF models and pointed out that industrial and agricultural activities were the main sources of soil heavy metal pollution in the Yunnan Pb–Zn mining area. Chen et al.^24,25^ reached a similar conclusion and pointed out that PMF could achieve finer identification of the pollution sources. Western Yunnan, China, is globally recognized for its widespread high geological background levels of heavy metals in soils, particularly Cd, Pb, and As. This region is characterized by a combination of natural and anthropogenic pollution sources, a unique distribution of geological sources, and a wide variety of human activities, which makes it difficult for a single model to comprehensively assess the contributions of different sources^26,27^. Although both APCS-MLR and PMF have been successfully applied in other areas, their joint application has not yet been reported for complex farmland soils under high-background conditions in western Yunnan.This highlights the need for more refined analytical approaches to understand pollution sources in such regions, and provides the rationale for applying a combined model framework in this study^28,29^.

In this study, we analyzed and evaluated the distribution characteristics of heavy metals and their comprehensive pollution in contaminated cropland under the combined influence of high geological background values for heavy metals and anthropogenic pollution in western Yunnan, China. The APCS-MLR and PMF models were used to investigate the sources and contributions of heavy metals, with the aim of providing a scientific basis for the safe utilization of contaminated arable land under the combined influence of high geological background values for heavy metals and anthropogenic pollution in western Yunnan, China.

## Materials and methods

### Overview of the study area

**Fig. 1:**
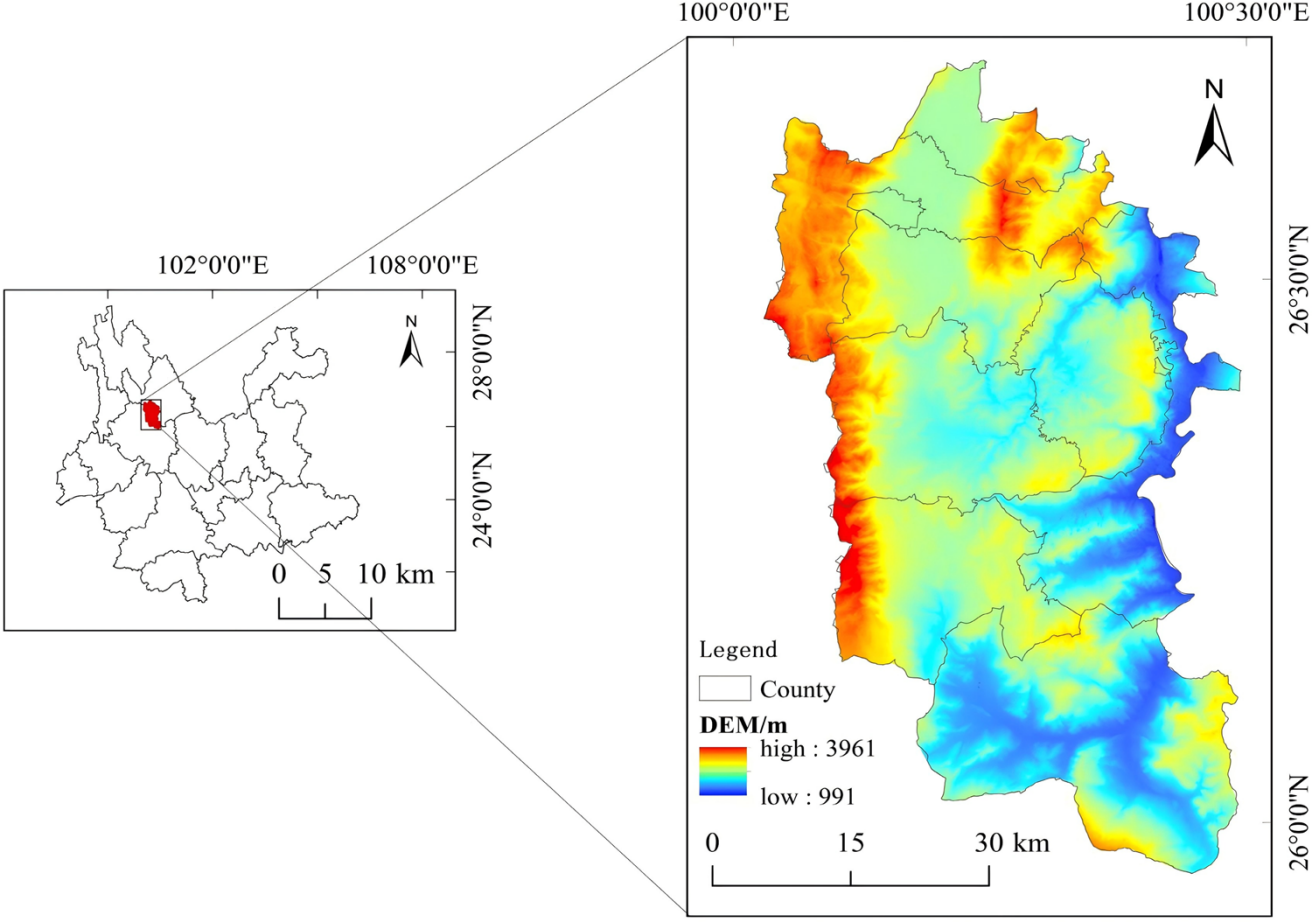
Overview map of the study area. National Catalogue Service for Geographic Information of China, National county-level administrative boundaries shapefile data (https://www.webmap.cn/main.do?method=index); Geospatial Data Cloud, 30-m resolution digital elevation data (https://www.gscloud.cn/). The map was created using ArcGIS 10.8 (Environmental Systems Research Institute, Redlands, CA, USA; https://www.esri.com/).

**Fig. 2:**
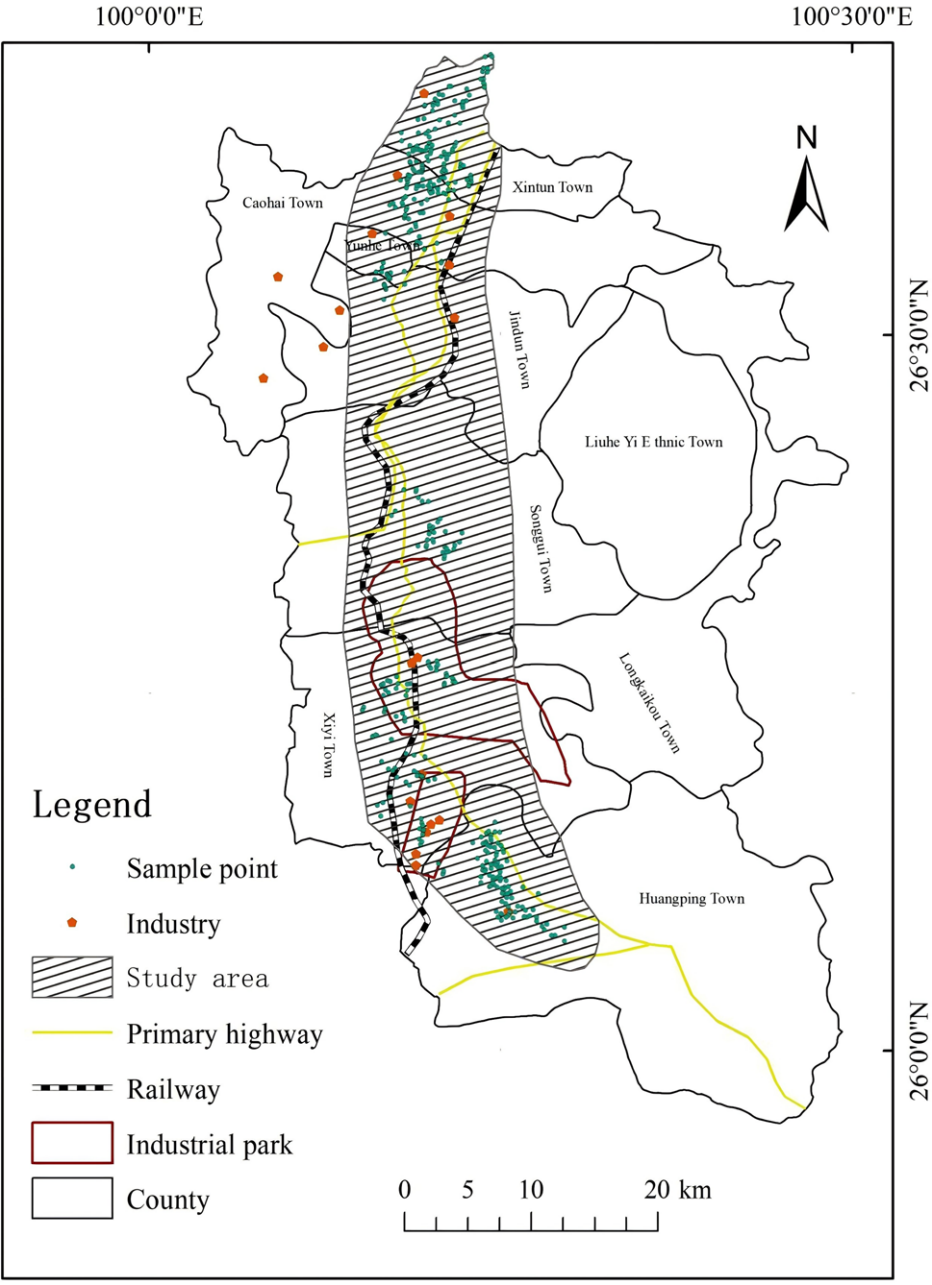
Map showing the distribution of the sampling points. National Catalogue Service for Geographic Information of China, National County-level Administrative Boundaries Shapefile Data (https://www.webmap.cn/main.do?method=index). The map was created using ArcGIS 10.8 (Environmental Systems Research Institute, Redlands, CA, USA; https://www.esri.com/).

The study area is located in the mountainous western Yunnan region of Yunnan Province, China (100°01′–100°29′ E, 25°57′–26°42′ N) (Fig. 1). This area has diverse geomorphological characteristics and is dominated by mountains and hills. The predominant soil type in the area is red soil. The region has a subtropical monsoon climate, with an average annual temperature of 14.7 °C, an average annual precipitation of 939.7 mm, and an mean elevation of 2696 m. The study area is rich in mineral resources, with 18 types of minerals and 12 deposits (mountains) discovered, and is an important mineral resource-rich area in western Yunnan. High-quality manganese-rich ores, rock-gold ores, and bauxite are the primary resources. The area covered by mining, beneficiation, smelting, metallurgical processing, and the production and processing of mineral resources has gradually grown. With high-quality manganese-rich ore and bauxite as the core, it has gradually formed a complete mining industry chain covering mining, ore dressing, smelting, and comprehensive utilization of resources. Long-term mineral mining and smelting activities have been provided the backbone of local economic development, though it has simultaneously impacted the surrounding ecological environment and soil quality. Through data collection, investigation, and monitoring analysis, it was found that there were 78,021.32 acres of contaminated arable land in this area, most of which was in seven townships (Fig. 2).

### Sample collection and processing

The distribution of the sampling points is shown in Fig. 2. Cropland soils surrounding the contaminated sites were selected as representative sampling areas. At each sampling site, five locations were selected for soil sampling within the 0 ~ 20 cm tillage layer using the double-diagonal five-point sampling method. The five sub-samples were mixed into a homogeneous sample immediately after sampling, and a total of 400 soil samples were collected. In the laboratory, the samples were air-dried at room temperature, and foreign materials such as gravel and film were removed. The samples were ground and passed through a 100-mesh nylon sieve, and sealed in polyethylene self-sealing bags prior to testing.

### Project measurement and methodology

The pH values of the soil samples were determined according to the Determination of Soil pH standard (NY/T 1377-2007), and the heavy metal contents were analyzed according to the following standard methods: Cd and Pb were analyzed according to the Determination of Lead and Cadmium in Soil Quality Graphite Furnace Atomic Absorption Spectrophotometric Method (GB/T 17141-1997); Cr, Cu, Zn, and Ni were analyzed according to the Determination of Copper, Zinc, Lead, Nickel, and Chromium in Soil and Sediment via Flame Atomic Absorption Spectrophotometry (HJ 491-2019); as was analyzed according to the Determination of Arsenic in Soil via Atomic Fluorescence Spectrometry (HJ 694-2014); and Hg was analyzed according to the Determination of Mercury in Soil via Cold Atomic Absorption Spectrophotometry (Cold AAS) (HJ 597-2011). The analyses were performed using a Thermo Scientific iCE 3000 AAS atomic absorption spectrometer. All of the reagents were of superior purity, and the water used was ultrapure water. Quality control was strictly carried out using blank samples, parallel samples, and spiked recovery samples. The correlation coefficients of the standard curves of the elements were greater than 0.999, the relative standard deviations between the parallel samples were less than 10%, and the recoveries of the spiked samples were 80% ~ 110%, demonstrating the accuracy and reliability of the data.

### Evaluation method

The ground accumulation index (Igeo)^26,30^ was used to comprehensively evaluate the soil heavy metal pollution. The Igeo is mainly used to reflect the enrichment level of individual heavy metals in the soil. All of the reference values in this study were selected from the background values of soil in Yunnan Province^4,31^.

### Source resolution models

#### APCS-MLR modeling

The APCS-MLR model is a pollutant source analysis method based on PCA^32^, and has been widely used in studies of soil heavy metal pollution^33^. It first extracts potential components via PCA, then converts factor scores into absolute principal component scores (APCSs), and finally uses multiple linear regression (MLR) to quantify the contribution of each source, with pollutant concentrations as the dependent variable and APCSs as independent variables. The number of components is typically determined by eigenvalues (> 1) or scree plot, and model performance is evaluated using *R*^2^ and significance tests.

#### PMF model

The PMF model, recommended by the U.S. EPA^34^, is based on factor analysis and decomposes a matrix of pollutant concentrations to identify source profiles and their contributions^35^. The number of factors is determined by Q-value comparison, residual analysis, and the interpretability of results. FPEAK values are adjusted to reduce rotational ambiguity. Uncertainties are estimated from detection limits and analytical errors and input into the model. Model performance is assessed by correlation between observed and predicted values, signal-to-noise ratios (S/N), and bootstrap or DISP tests.

### Data processing and analysis

Excel was used to carry out the statistics and calculations of the heavy metal contents and ground accumulation index. ArcGIS 10.8 was used to produce the overview map of the study area and the spatial distribution maps of the heavy metals. Origin 2024 was used to plot the ground accumulation index. SPSS 26 was used to conduct the principal component and correlation analyses; and EPA PMF 5.0 was used to analyze the heavy metal pollution sources.

## Results and analysis

### Characteristics of soil heavy metal contents

The environmental quality of the soil in the study area is presented in Table 1, which shows that the soil in the study area is generally neutral. Eight heavy metal elements were within the medium–high intensity variation range. Hg and Pb were within the high intensity variation range, and the order of the values of the coefficients of variation are as follows: Pb > Hg > Cd > Zn > As > Cu > Cr > Ni. Compared with the background values of the soil heavy metals in Yunnan Province, the average contents of the eight heavy metals were higher than the background values of the soil, i.e., 1.26 to 6.01 times higher. The pollution levels of the eight heavy metals in the soils in the study area were evaluated using the soil pollution screening values in the Soil Environmental Quality Risk Control Standards for Soil Pollution on Agricultural Land (Trial) (GB 15618-2018) as reference values (Table 2). The heavy metal contents of all of the soil samples collected from the study area exceeded the standard values, except for Hg, and their exceedance rates were Cd (80.0%), Cu (52.0%), Cr (36.0%), As (35.5%), Pb (30.0%), Ni (29.5%), and Zn (23.8%).

**Table 1:** Statistics of heavy metal contents of soil in the study area.

Element	Maximum (mg/kg)	Minimum (mg/kg)	Mean (mg/kg)	Median (mg/kg)	Standard deviation	Coefficient of variation	Soil background values in Yunnan Province
Cu	468.00	24.10	123.54	89.75	78.89	0.64	46.3
Zn	1310.00	45.30	232.27	148.00	196.56	0.85	89.7
As	177.00	2.70	26.38	20.75	19.58	0.74	18.4
Hg	1.98	0.02	0.14	0.10	0.14	1.00	0.06
Cd	9.95	0.16	1.26	0.70	1.26	0.99	0.22
Pb	3780.00	14.50	244.18	51.80	442.39	1.81	40.6
Ni	337.00	20.30	115.89	115.00	54.56	0.47	42.5
Cr	682.00	54.10	205.36	200.00	99.70	0.49	65.2
pH	8.60	4.08	7.17	7.49	0.91	0.13	5.7

**Table 2:** Frequency and proportion of soils exceeding risk screening values.

Element	Number of exceedances of risk screening values				
	pH ≤ 5.5	5.5 < pH ≤ 6.5	6.5 < pH ≤ 7.5	pH > 7.5	Total
Cu	20 (76.9)	52 (7.5)	42 (38.5)	94 (47.7)	208 (52.0)
Zn	7 (26.9)	10 (14.7)	19 (17.4)	59 (30.0)	95 (23.8)
As	1 (3.8)	4 (5.9)	44 (40.4)	93 (47.2)	142 (35.5)
Hg	0 (0.0)	0 (0.0)	0 (0.0)	0 (0.0)	0 (0.0)
Cd	25 (96.2)	68 (100.0)	107 (98.2)	120 (61.0)	320 (80.0)
Pb	13 (36.1)	27 (39.7)	28 (25.7)	52 (26.4)	120 (30.0)
Ni	15 (57.7)	30 (44.1)	59 (54.1)	14 (7.1)	118 (29.5)
Cr	6 (23.1)	22 (3)	56 (51.4)	60 (30.5)	144 (36.0)
pH	26	68	109	197	400

### Characterization of spatial distributions of heavy metals in soil

**Fig. 3:**
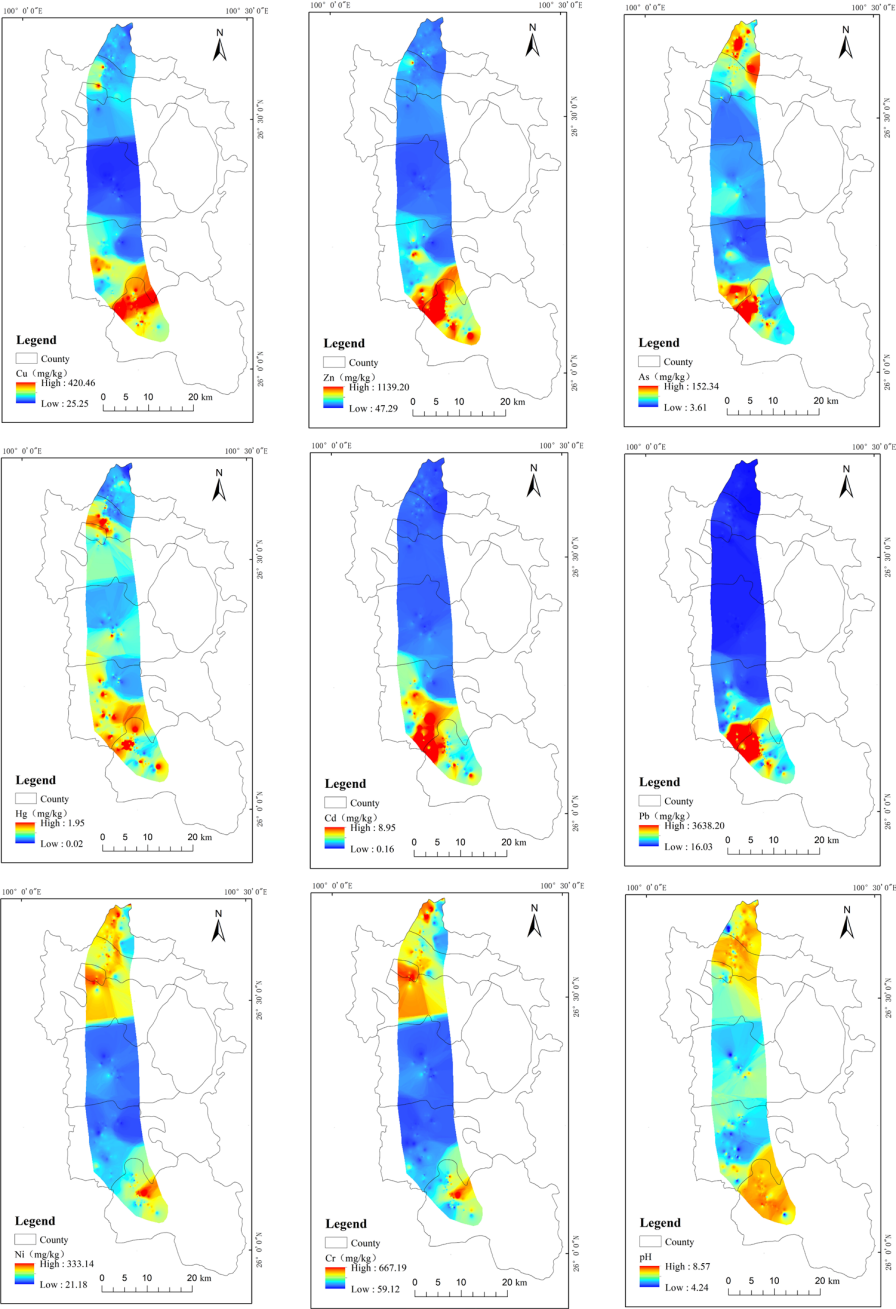
Spatial distributions of heavy metal contents and pH in arable soil. National Catalogue Service for Geographic Information of China, National County-level Administrative Boundaries Shapefile Data (https://www.webmap.cn/main.do?method=index). The map was created using ArcGIS 10.8 (Environmental Systems Research Institute, Redlands, CA, USA; https://www.esri.com/).

Inverse distance weighted (IDW) interpolation was used to map the spatial distributions of the heavy metal contents. By analyzing the natural turning points and characteristic points, the heavy metal contents were classified into clusters with similar properties to maximize the differences between the classes (Fig. 3). Cd, Pb, Zn, and Cu exhibited similar piecewise enrichment characteristics in the study area, and the high-value areas were mainly concentrated in the southern part of the study area, demonstrating that human activities had a significant influence on the distributions of the soil heavy metals in the areas close to the industrial concentration areas and near the main transportation routes. Cr and Ni exhibited similar piecewise enrichment, and there was a strong spatial correlation between the distributions of As, Hg, and pH. In the high-value heavy metal content areas, the pH value was correspondingly higher. Based on analysis of the coefficients of variation, the spatial distribution patterns of these elements in the study area are similar, indicating that the distributions of these elements are influenced by similar factors.

### Evaluation of soil land accumulation index in the study area

**Fig. 4:**
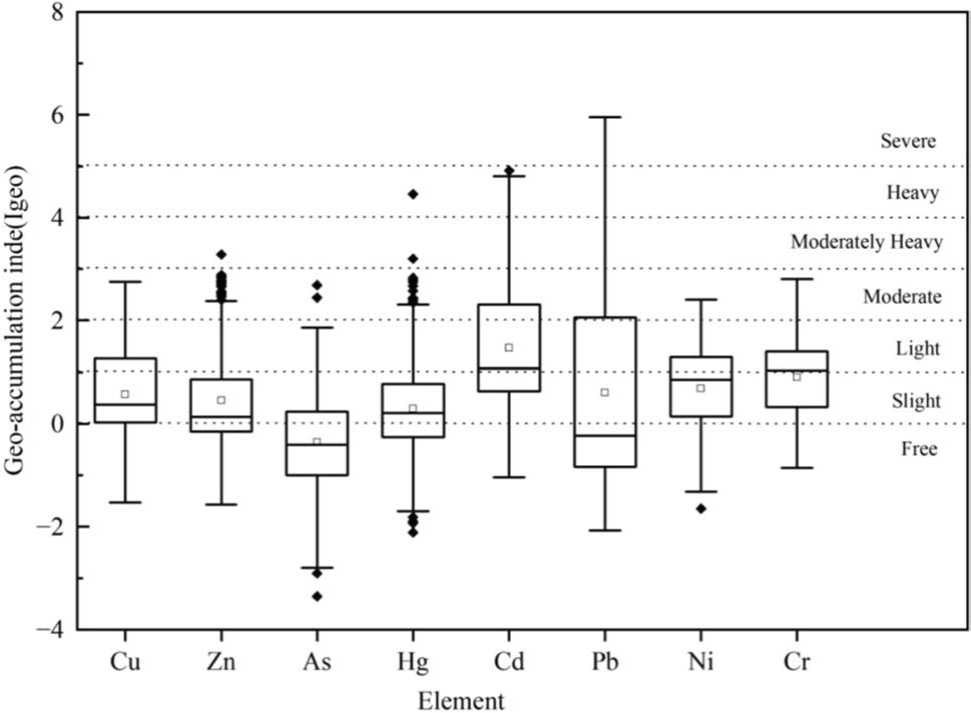
Box plot of land accumulation index.

**Fig. 5:**
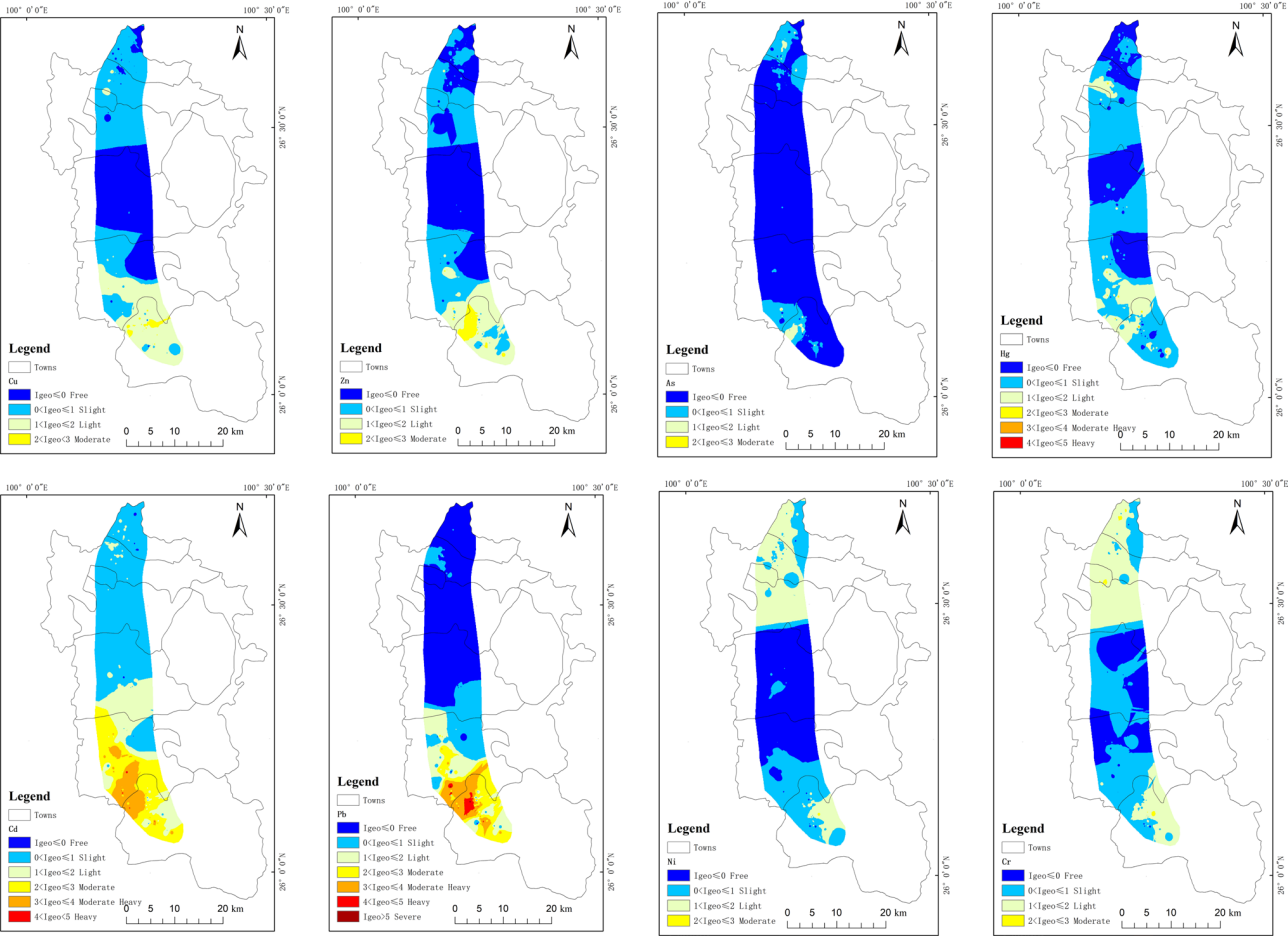
Spatial distributions of the land accumulation index values of the heavy metals in arable soils. National Catalogue Service for Geographic Information of China, National County-level Administrative Boundaries Shapefile Data (https://www.webmap.cn/main.do?method=index). The map was created using ArcGIS 10.8 (Environmental Systems Research Institute, Redlands, CA, USA; https://www.esri.com/).

The results of the evaluation of the soil geoaccumulation index of the cultivated land in the study area are shown in Fig. 4. The results show that the ranges of the land accumulation indices for Cu, Zn, As, Hg, Cd, Pb, Ni, and Cr were − 1.5–2.8, − 1.6–3.3, − 3.4–2.7, − 2.1–4.5, − 1.0–4.9, − 2.1–6.0, − 1.7–2.4, and − 0.9–2.8, with mean values of 0.6, 0.5, − 0.4, 0.3, 1.5, 0.6, 0.7, and 0.9, respectively. The order of the pollution degrees of the elements was Cd ≥ Cr ≥ Ni ≥ Pb ≥ Cu ≥ Zn ≥ Hg ≥ As. The spatial distributions of land accumulation indices for the eight heavy metals exhibited different degrees of variations (Fig. 5). Among then, Cd and Pb had the highest land accumulation pollution indices, reaching 4.9 and 6.0, respectively, indicating that there was a significant anthropogenic pollution source in localized areas^36^. These areas were mainly concentrated in the southern part of the study area and near the industrial and transportation areas, suggesting that industrial activities and transportation emissions were the key factors influencing the accumulation of these heavy metals. Comparatively speaking, the geoaccumulation index of As was lower, indicating that the degree of accumulated pollution was slight and was closer to the natural background value. The overall level of the soil heavy metal accumulation in the study area was mainly slight to mildly polluted, and it did not reach heavy or serious pollution. The pollution level was greater in the southern part of the study area than in the northern part of the study area.

### Pollution source analysis modeling

#### Correlation analysis

**Fig. 6:**
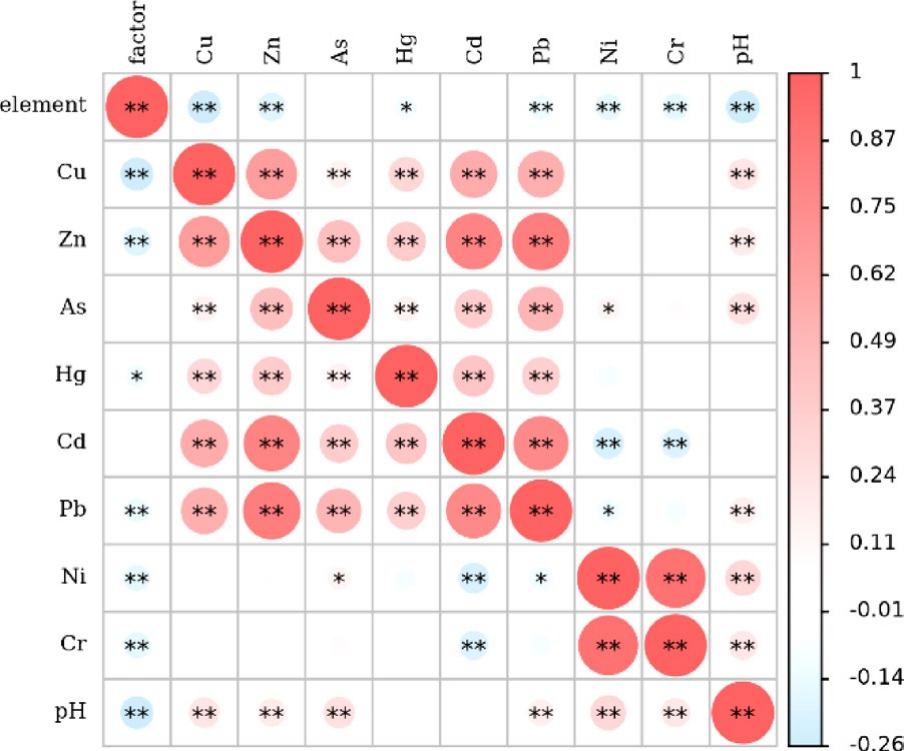
Results of correlation analysis of heavy metal content of soil.

Pearson correlation analysis was performed on the contents of the eight heavy metal elements. The results are shown in Fig. 6. The results showed that many of the heavy metal elements and the pH exhibited significant correlations, indicating that they had common sources and similar migration and transformation processes. Cu, Zn, Cd, and Pb exhibited significant positive correlations, indicating that these four elements had similar input pathways, such as industrial emissions, mining activities, and/or agricultural inputs. Additionally, the correlations of As with Hg, Cd, and Pb further revealed the contributions of deposition and/or anthropogenic disturbances to the accumulation of these heavy metals. In addition, the independence of Ni and Cr indicated that they originated from natural geochemical processes rather than from significant exogenous pollution inputs. In addition, the pH, as an important environmental factor, profoundly affected the morphology, solubility, and transport behaviors of the heavy metals in the soil.

#### APCS-MLR modeling

Multiple linear regression analysis of the eight heavy metal elements revealed that the Bartlett’s sphericity test companion probability was 0, which was lower than the significance level of 0.05, and the Kaiser–Meyer–Olkin (KMO) test statistic value was 0.753, indicating that the sample data for the study area was suitable for PCA^37^. Through PCA, three principal components were extracted (Table 3). The first principal component (F1) had a variance contribution rate of 44.78%, and it explained the largest proportion of the variance in soil heavy metal distributions, corresponding to high concentrations or strong symbiosis of the heavy metal fractions. The second principal component (F2) and the third principal component (F3) contributed 24.53% and 11.22%, respectively, with a cumulative variance contribution of 80.53%, which explained most of the information about the heavy metal pollution.

**Table 3:** Principal component analysis results: rotated component loadings and variance explained.

Components	Initial eigenvalues			Extracted load sum of squares			Rotated sum of squared loadings		
	Total	%Var	%Cum	Total	%Var	%Cum	Total	%Var	%Cum
F1	3.59	44.78	44.78	3.58	44.78	44.78	2.68	33.55	33.55
F2	1.97	24.53	69.31	1.96	24.53	69.31	1.99	24.87	58.43
F3	0.90	11.22	80.53	0.90	11.22	80.53	1.77	22.10	80.53
F4	0.75	9.39	89.91						
F5	0.35	4.43	94.34						
F6	0.22	2.76	97.09						
F7	0.14	1.78	98.87						
F8	0.09	1.13	100.00						

Note: “Initial eigenvalues” indicate the variance explained by each principal component prior to rotation. “Extracted load sum of squares” refers to the variance explained by the extracted components before rotation. “Rotated sum of squared loadings” eflects the redistributed variance contributions after Varimax rotation. %Var denotes percentage of variance explained by each component; %Cum represents cumulative explained variance. Only components with eigenvalues > 1 were retained, based on the Kaiser criterion.

The explanatory degree of F1 was significantly higher than those of the other principal components, and the heavy metals with higher loadings were Hg, Cu, Zn, Cd, and Pb. Lv et al. (2013) and Wang et al. (2021) showed that when Cd, Pb, and Zn were classified into the same principal component, they were mainly affected by anthropogenic sources, and the contribution of Hg was the highest (66.99%)^38,39^. Studies have also shown that Hg is affected by multiple factors, such as soil-forming matrices, industrial emissions, transportation, and agriculture, and that bituminous coal has a significant effect on its accumulation in soil^40,41^. In addition, transportation exhaust, dust, and particles can all contribute to the increase in soil Hg after atmospheric deposition. According to the spatial distribution characteristics and correlation analysis of the heavy metals, Cu, Hg, Zn, Cd, and Pb were highly enriched and correlated in the southern part of the study area, and the pollution was particularly serious in the industrial parks and near the transportation routes, indicating that in the study area, these metals were greatly influenced by industrial and transportation sources. Based on the high value areas in the spatial distributions of these five elements and the distributions of the industrial zones and transportation routes, it was concluded that F1 was an industrial and transportation source.

The F2 source contributed the most to Cr and Ni, reaching 96.99% and 93.51%, followed by Cd (25.26%). Some studies have shown that Cr and Ni are mainly affected by the soil parent material and soil-forming processes^42,43^. The ground accumulation indexes and comprehensive pollution indexes of Ni and Cr in the study area did not indicate pollution, and their coefficients of variation were relatively small, further indicating that the Ni and Cr in the soil in the study area were less influenced by exogenous sources and mainly came from the parent material of soil formation processes. Therefore, we concluded that F2 was the parent material source.

Source F3 contributed the most to As, Zn, and Pb, with values of 51.75%, 26.18%, and 22.95%, respectively. According to the preliminary investigation, this area is rich in mineral resources, and there are non-ferrous metal resources such as gold, lead–zinc, and copper ores in the study area, which is an important mineral development zone in Yunnan Province. As is an accompanying element in gold ores. Based on the fact that the areas with high As, Zn, and Pb values overlapped with smelting plant locations, we concluded that F4 was a mining source.

Unknown source F4 mainly contributed to As, Pb, and Hg, with contribution values of 26.49%, 25.52%, and 19.86%, respectively, although these contribution values were not high. Hg and Pb are important components of pesticide fertilizers, can enter the soil through their application and become enriched on the soil surface^44^. The main land use type in the study area is arable land, suggesting that this part of the impact originates from agricultural activities; therefore, we conclude that F4 is an agricultural source.

**Fig. 7:**
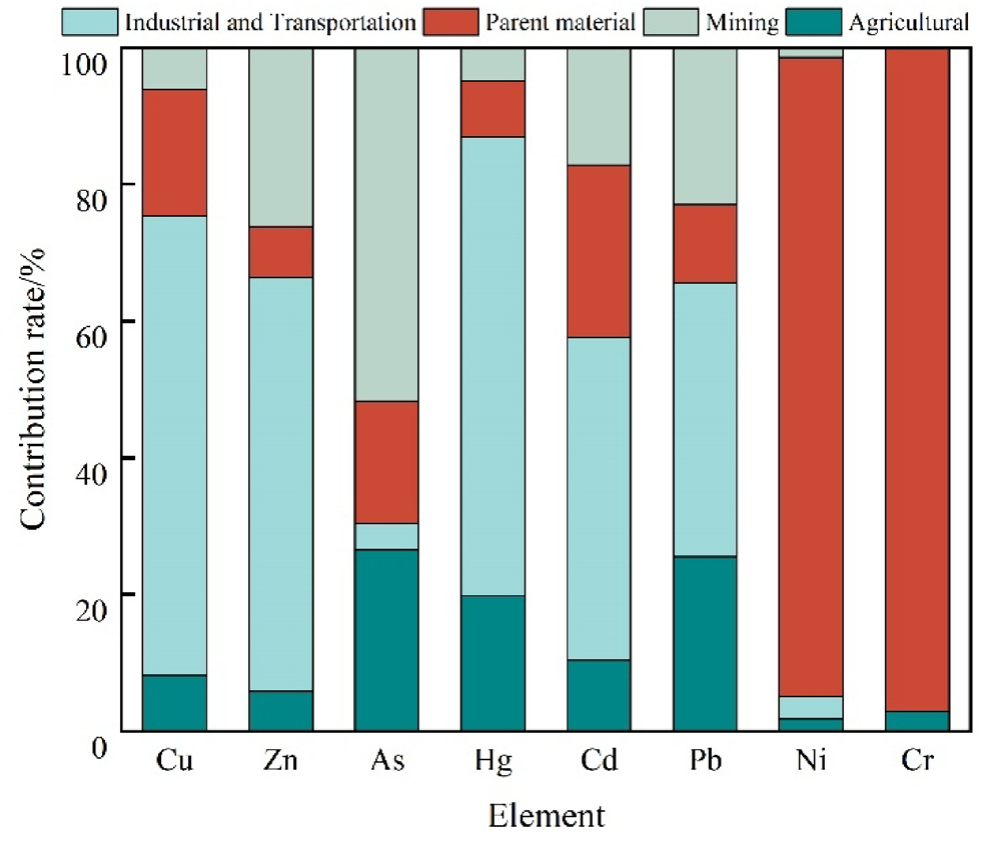
Contributions of pollution sources in the APCS-MLR model.

The APCS-MLR model was used to analyze the eight heavy metals via multiple linear regression (Fig. 7). The predicted values fit well with the measured values, and the results show that the *R*^2^ values of Cu, Zn, As, Hg, Cd, Pb, Ni, and Cr are 0.687, 0.867, 0.841, 0.497, 0.810, 0.838, 0.949, and 0.942, with an average *R*^*2*^ value of 0.804. The estimated/measured values (E/O) of all of the elements were close to 1, indicating that the APCS-MLR model analysis results have a high credibility. Notably, the *R*^*2*^ value of Hg was relatively moderate (0.497), which may be attributed to the discontinuous and nonlinear nature of agricultural Hg inputs, such as sporadic application of Hg-containing fertilizers or pesticides. The industrial and transportation activities contributed substantially to the Cu, Zn, Hg, Cd, and Pb pollution. The soil matrices had a significant influence on the Cr, Ni, and Cd pollution and were the main sources. The mining industry also had a large influence on the As, Zn, and Pb pollution.

#### PMF model

**Fig. 8:**
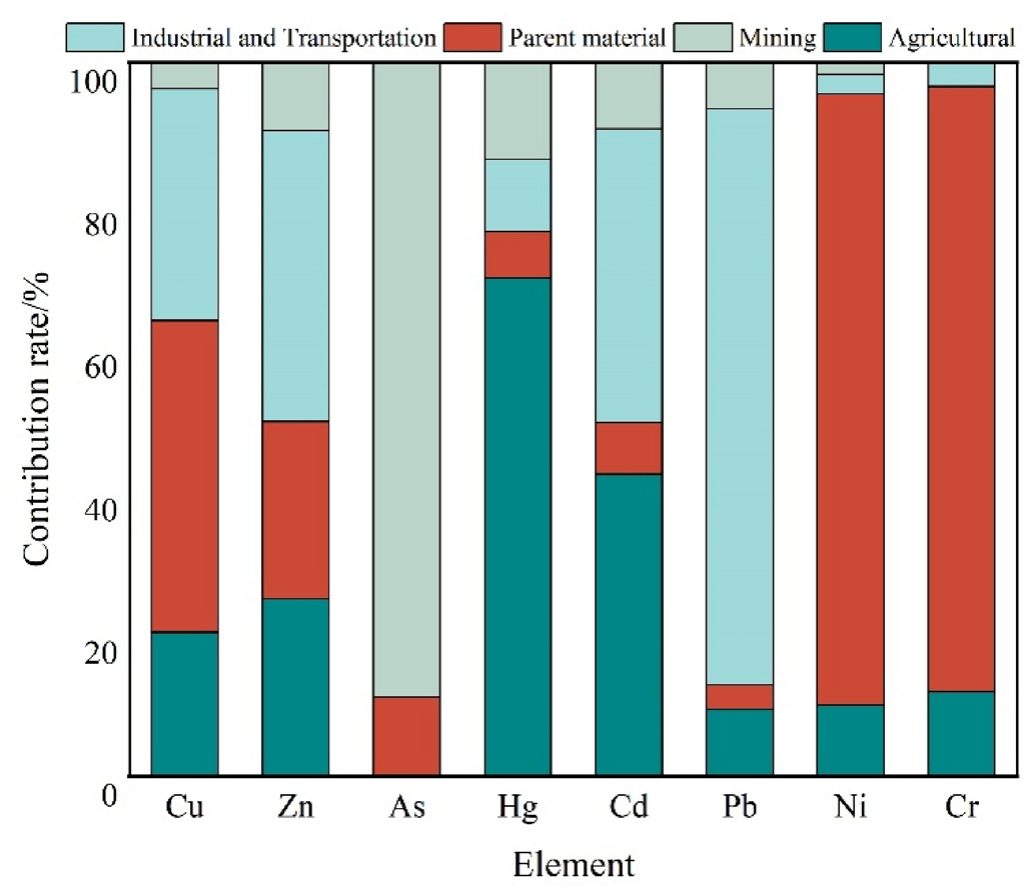
PMF model pollution source contributions.

In order to verify the reliability of the source analysis results of the APCS-MLR model, the PMF model was used to conduct quantitative source analysis of the eight heavy metals. Three to five factors were selected for random iterative operations, and four factors were finally determined to obtain the contributions of the four heavy metal pollution sources to each heavy metal (Fig. 8).

The elements with high loadings for Factor 1 were Pb, Cd, Zn, and Cu, which were mainly found in mining areas, transportation routes, and other areas with intensive human activities. This aligns well with the results of the APCS-MLR model analysis. The results of both models indicated that Factor 1 was an industrial and transportation source. Factor 2 contributed the most to Cr and Ni, which had small coefficients of variation. The APCS-MLR model results were highly consistent with the results of the APCS-MLR model, and both models indicated that Factor 2 was a parent material source. Factor 3 had the highest loading for As, which aligned with the fact that the geologic background of the study area was rich in mineral resources and is an important development zone for non-ferrous metals, such as gold, in Yunnan Province. This is essentially the same as the results from the APCS-MLR model, and both models indicate that Factor 3 is a mining industry source. Factor 4 had high loadings for Hg and Cd. Several studies have shown that fertilizers and pesticides often contain elements, such as Hg, Cd, and Pb, which can be indicative of agricultural activities such as the application of pesticides and fertilizers^45^. Thus, we concluded that Factor 4 was an agricultural source. The PMF model identifies the four sources of contamination of the eight heavy metals as industrial traffic, parent material, mining, and agricultural sources.

The results of the pollution source contribution analysis using the PMF model (Fig. 8) correspond well with the results of the correlation analysis (Fig. 6) and the APCS-MLR model analysis (Fig. 7). The industrial and transportation activities contributed more to the Cu, Zn, Hg, Cd, and Pb pollution. The soil matrix had an important influence on the Cr and Ni, which aligned with the independence of several elements in the correlation analysis and the natural sources corresponding to F2 in the APCS-MLR model. The mining sources had an influence on As, Hg, and Cd, which aligned with the analysis of F3 in the APCS-MLR model, and the fact that agricultural sources had a greater influence on Hg and Cd. This conclusion is reflected by the results presented in Fig. 6, Table 3, and Fig. 7, which show the main composition of the soil heavy metal pollution sources and their relative contributions in the study area.

## Discussion

### Universal analysis of heavy metal pollution characteristics and causes

This study reveals the combined factors behind heavy metal contamination in cropland soils of the western Yunnan high geological background region: both natural parent material contributions and anthropogenic activities have jointly shaped the contamination patterns. Similar to other geological regions with high background levels worldwide (e.g., the ophiolite region of Greece, the Deccan Plateau of India), elements such as Cr and Ni in the study area are primarily sourced from the parent material (with contribution rates > 90%), and their spatial distribution closely aligns with the weathering characteristics of the regional bedrock^46,47^. In contrast, elements such as Cd, Pb, and Cu exhibit significant enrichment (with exceedance rates ranging from 30 to 80%), with Cd and Pb concentrations reaching 6.01 and 4.2 times their background values, respectively, indicating that human activities are the primary driving force behind the intensification of pollution^10^. Spatial analysis further reveals that the accumulation coefficients of Cd and Pb around industrial areas and transportation corridors are as high as 4.9 to 6.0, with pollution patterns similar to those observed in typical mining areas worldwide, such as the Mount Isa Pb–Zn mining region in Australia^47,48^, and the contribution of Pb and Zn from transportation emissions in the Mississippi River Basin in the United States^25,49^, collectively confirming the significant role of industrial and transportation sources. Notably, the pollution characteristics of As in western Yunnan are distinctive: despite a low geocumulative index (− 0.4), areas of high As concentration overlap significantly with the distribution of gold mining belts, suggesting a synergistic effect between geological background and mining activities. This type of “implicit high background superimposed with anthropogenic disturbance” pollution pattern is widely observed in lateritic gold mining areas across Southeast Asia (e.g., Laos, Myanmar)^50^, indicating that the “geological-anthropogenic dual-source coupling” analytical framework proposed in this study holds substantial potential for regional application.

### Advantages of multi-modeling for analyzing complex pollution areas

The combined application of the APCS-MLR and PMF models effectively addresses the limitations associated with using a single model to analyze complex pollution sources. ① Complementarity in source apportionment: Both models yielded consistent results, indicating that the combined contribution of Cd and Pb ranged from 58 to 63%. This is in close agreement with the emission inventory data from lead–zinc smelting areas in western Yunnan, where Cu, Zn, and Cd collectively account for more than 50% of total emissions^18^. APCS-MLR identifies the direct contribution of industrial and traffic sources to Cd and Pb through the principal component loadings (*R*^2^ = 0.84). In contrast, the PMF model captures the more complex and indirect inputs of agricultural sources to Hg, with an estimated contribution of 19%–26% derived from factor decomposition. The combined use of both models provides a comprehensive view of the continuous and discrete nature of pollution sources^51^. ② Reduction of Geological Background Interference: To address the issue of “false-positive contamination” from Cr and Ni in high-background areas, the dual-model approach has been validated through both coefficient of variation analysis (CV < 15%) and elemental correlation networks (*r* = 0.92), successfully removing the interference from parent material sources on the contamination index. This approach can be applied to similar geological regions, such as the Iron Quadrangle in Brazil and the Bushveld Complex in South Africa^52^. ③ Optimization of Agricultural Source Quantification: The PMF model captures the nonlinear relationship between Hg and fertilizer application intensity (*R*^2^ = 0.47), addressing the limitations of APCS-MLR in resolving discrete sources. This is particularly important in cases like Hg, where the APCS-MLR model showed only moderate fit (*R*^2^ = 0.497), likely due to irregular agricultural inputs. The improved resolution offered by PMF provides a valuable methodological reference for similar agriculture–mining complex regions. ④ Accuracy of Policy Response: Model analysis indicates that industrial and transportation sources contribute 58% (APCS-MLR) to 63% (PMF) of the Cd pollution, significantly higher than the 35% contribution observed in typical agricultural areas of southern China^18^. This suggests that western Yunnan should prioritize targeted strategies such as “cleaner production reform in key industries + interception of particulate matter from transportation emissions” rather than simply applying agronomic control measures used in other regions. In addition, Cd also showed a moderate contribution (25.26%) from the parent material source (F2), indicating that geogenic background still plays a non-negligible role. This reflects the complex dual-source characteristics of Cd in the region, suggesting the need for differentiated control measures that consider both anthropogenic and natural sources.

### Decision-support for differentiated management of safe use of agricultural soils

This “pollution characterization–source analysis–risk zoning” framework supports the development of refined soil management strategies tailored to varying contamination levels and specific agricultural land use patterns, including crop sensitivity and planting objectives. ① High Geological Background Control Zone (Cr and Ni Enrichment Zones): Implement low-accumulation crop cultivation and pH regulation to prevent the activation of parent material and its release^53^. ② Compound Pollution Remediation Zone (Cd, Pb Exceedance Zones): Focus on structural adjustments, while adopting plant passivator and blocking technologies to mitigate risks to human health. Additionally, source interception should be applied to industrial and transportation sources^54^. ③ Compound Risk Early-Warning Zone (Cd and Pb Compound Pollution Zones): Establish a dynamic monitoring network to track real-time changes in soil and environmental quality, and prioritize the closure or upgrading of non-compliant smelting enterprises to cut off major anthropogenic pollution sources. This hierarchical control strategy has been validated in typical karst regions of Southwest China and has been shown to reduce pollution migration risks in areas with complex geological backgrounds and compound pollution^55^.

## Conclusions

1. The soil in the study area was overall neutral, and the average contents of the eight heavy metals, namely, Cu, Zn, As, Hg, Cd, Pb, Ni, and Cr, ranging from 1.26 to 6.01 times the background values in Yunnan Province. Among them, Cd and Pb pollution was relatively severe, with concentrations in 80% and 30% of the samples, respectively, exceeding the risk screening values for agricultural land (GB 15618-2018).
2. The spatial distributions of heavy metals showed considerable heterogeneity. Based on the cumulative geoaccumulation index, which integrates metal enrichment and potential ecological risk, Cd and Pb exhibited the highest levels, indicating stronger anthropogenic influence and higher management priority. In contrast, Hg had the lowest index, suggesting limited accumulation and relatively low environmental concern.
3. This study demonstrated that heavy metal contamination in the study area was primarily attributed to industrial and traffic-related emissions (accounting for 58%–63% of Cd and Pb) and to geogenic sources, with parent materials contributing over 90% of Cr and Ni. A nonlinear contribution of agricultural sources to Hg was identified by the PMF model, likely resulting from sporadic applications of Hg-containing agrochemicals. These findings highlight the necessity of strengthened regulation of agricultural inputs, the promotion of low-Hg alternatives, and targeted environmental monitoring in agricultural zones. The integrated use of dual-source apportionment models significantly improved source differentiation and clarified the respective roles of anthropogenic and natural influences, thereby providing a scientific basis for source-specific remediation and stratified management of contaminated croplands.

## Data Availability

The datasets used and/or analysis during the current study available from the corresponding author on reasonable request.
